# Single-Piece All-Solid-State Potential Ion-Selective Electrodes Integrated with Molecularly Imprinted Polymers (MIPs) for Neutral 2,4-Dichlorophenol Assessment

**DOI:** 10.3390/ma12182924

**Published:** 2019-09-10

**Authors:** Samar Ezzat, Mona A. Ahmed, Abd El-Galil E. Amr, Mohamed A. Al-Omar, Ayman H. Kamel, Nagy M. Khalifa

**Affiliations:** 1 Department of Chemistry, Faculty of Science, Ain Shams University, 11566 Cairo, Egypt; 2 Chemistry Department, College for Women, Ain Shams University, Heliopolis, 11751 Cairo, Egypt; 3 Pharmaceutical Chemistry Department, Drug Exploration & Development Chair (DEDC), College of Pharmacy, King Saud University, Riyadh 11451, Saudi Arabia; 4 Applied Organic Chemistry Department, National Research Center, 12622 Giza, Egypt

**Keywords:** solid-contact ISEs, molecularly imprinted polymers (MIPs), chlorophenols, 2,4-dichlorophenol, neutral response mechanism.

## Abstract

A novel single-piece all-solid-state ion-selective electrode (SC/ISE) based on carbon-screen printed is introduced. Polyaniline (PANI) is dissolved in a membrane cocktail that contains the same components used for making a conventional ion-selective polyvinyl chloride (PVC) matrix membrane. The membrane, having the PANI, is directly drop-casted on a carbon substrate (screen-printed-carbon electrode). PANI was added to act as an intermediary between the substrate and the membrane for the charge transfer process. Under non-equilibrium sensing mechanism, the sensors revealed high sensitivity towards 2,4-dichlorophenol (DCP) over the linearity range 0.47 to 13 µM and a detection limit 0.13 µm. The selectivity was measured by the modified separate solution method (MSSM) and showed good selectivity towards 2,4-DCP over the most commonly studied ions. All measurements were done in 30 mm Tris buffer solution at a pH 5.0. Using constant-current chronopotentiometry, the potential drift for the proposed electrodes was checked. Improvement in the potential stability of the SPE was observed after the addition of PANI in the sensing membrane as compared to the corresponding coated-wire electrode (membrane without PANI). The applicability of the sensor has been checked by measuring 2,4-DCP in different water samples and the results were compared with the standard HPLC method.

## 1. Introduction

Chlorophenols, a class of organic pollutants, have recently drawn considerable attention. These organochlorine compounds have been classified as persistent organic pollutants (POPs) as well as endocrine disrupting compounds (EDCs) [1,2,3]. Due to their constancy in aquatic environments, they can be released to either surface or ground waters as good as bottom sediments. 2,4-DCP has been widely applied in the yield of various herbicides, pesticides, preservatives, and plant growth regulators [4]. Most of the chlorinated phenols can be found and are accumulated in the human body through the food cycle. They can cause felled effects such as faintness, itch, anemia, and cancer risk at extremely low concentration levels [3,5]. After inhalation of 2,4-DCP, the respiratory tract is irritated and this is harmful to the liver, kidneys and organs forming blood [6,7,8,9]. Long exposure to 2,4-DCP can cause serious effects to the respiratory system in addition to permanent sight failure or blindness [7,8,9,10]. The European Union and US Environmental Protection Agency (EPA) have regulated its maximum allowable concentration in water at 0.5 µg/L [11]. Therefore, it is important to introduce fast, rigorous, precise, and cost-effective analytical methods to identify and assess this pollutant. 

Recently, different reported methods such as HPLC [12], GC/MS [13,14], chemiluminescence [15,16], capillary electrophoresis [17], and UV spectrophotometry [18] have been established for the assessment of 2,4-DCP. Chlorophenol compounds are electrochemically active because of the phenolic –OH group. So, some electrochemical sensors for the assessment of 2,4-DCP have been also reported [19]. These electrochemical sensors showed some excellent features such as fast response and high sensitivity. However, they exhibit poor selectivity because they are limited to distinguishing 2,4-DCP and other chlorophenols. This can be assigned to the lack of special recognition design.

Molecularly imprinted polymers (MIPs) when integrated with electrochemical sensors have picked up a great attention because of their high selectivity and sensitivity [20,21,22]. In addition, these artificial receptors have high robustness, good stability towards high temperatures and pressures, high resistance towards chemical changes, cost-effective, and can be re-used without remarkable deterioration [23]. Recently, integration of MIPs with ISEs revealed a success in organics detection [24,25,26]. Only very few potentiometric sensors for 2,4-DCP determination were reported recently [24,27]. Doping of quaternary ammonium salt in polymeric membranes revealed a super-Nernstian response towards electrically neutral phenolic compounds at neutral pH values. These unexpected anionic responses are due to the net movement of H^+^ ions to the aqueous medium coming from the membrane. These H^+^ were induced after the complexation of neutral phenols with the quaternary ammonium salt present in the sensing membrane [20,23,28]. 

Nowadays, solid-contact ion-selective electrodes SC/ISEs have drawn important attention for environmental monitoring. In the beginning, they were designed in the form of planar configuration called “screen printed electrodes (SPEs)”. The ion-sensing membranes (ISMs) were drop-casted on the conductive substrate in the platform [29]. However, the ill-defined interface between the ISM and the conductive substrate often causes a drift for the electrode potential [30,31]. Thus, much study has been concentrated on introducing new solid-contact transducers to enhance the performance characteristics of the electrodes. One of these important transducers are conducting polymers (CPs), which are characterized by their high electronic and ionic conductivity [32]. A minimal potential drift can be achieved by inserting the solid-contact material between the sensing membrane and electronic substrate. Conducting polymers (CPs) have redox buffer capacity through their oxidation/reduction. Therefore, they provide an effective ion-to-electron transduction. The response mechanism using these CPs was previously explained by several works [33,34]. The implementation of solid contact materials in the electrode can be done in two ways: (i) modification of the substrate of the electrode as in the case of redox active monolayers and carbon-based materials [35,36,37,38,39,40]. (ii) By directly dissolving the solid-contact transducer in the sensing membrane cocktail and then is drop-cast on the substrate electrode (i.e., so called “Single-Piece-ISEs”) [41]. The later provides good compatibility between the transducer and the conductive substrate of the electrode [41]. 

In this work, novel single-piece all solid-contact screen-printed sensors assigned with MIP beads for neutral 2,4-DCP as a persistent organic pollutants (POPs) are prepared and characterized. Polyaniline (PANI) film is used as a solid contact transducer. The MIP beads are used as selective receptors for 2,4-DCP and incorporated into the sensing membrane. The presented screen-printed platforms revealed a high sensitivity for the assessment of 2,4-DCP. 

## 2. Materials and Methods 

### 2.1. Materials and Instruments

“2,4-dichlorophenol (DCP), 2-chlorophenol (CP), methyl methacrylate (MMA), ethylene glycol dimethacrylate (EGDMA), benzoylperoxide (BPO) and acetonitrile were obtained from Sigma-Aldrich (St. Lois, USA). High molecular weight poly (vinyl chloride) (PVC), dioctyl phthalate (DOP) and tridodecylmethylammonium chloride (TDMACl) were obtained from Fluka AG (Buchs, Switzerland). Tetrahydrofuran (THF) was distilled before use. Aqueous solutions of 2,4-DCP were prepared with freshly de-ionized water (18.2 MΩ cm specific resistance). 30 mm Tris buffer solution of pH 5.0 was used for all subsequent experiments to ensure that 2,4-dichlorophenol is in its de-protonated form”. 

“Fourier-transform infrared spectroscopy (FT-IR) measurements were carried out using FT-IR spectrometer (Alpha II, Bruker ABCO, Cairo, Egypt) using the attenuated total reflection (ATR) technique. Impedance and chronopotentiometry measurements of the screen-printed electrodes (SPE) were measured using Metrohom potentiostat/galvanostat (Autolab, model 204) purchased from Metrohom Instruments (Herisau, Switzerland). A three-electrode configuration cell containing Silver/Silver chloride (3 M KCl) reference electrode and an auxiliary electrode made from platinum wire was employed. The spectra of impedance were recorded at open-circuit potential in 0.01 V 4-dichlorophenol solution with an excitation amplitude of 10 mV. The frequency range starts from 100 kHz to 0.1 Hz. Chronopotentiometry was done at a constant current of ±1 nA, applied to the working electrode for 60 s followed by a reversed current for another 60 s. The electrolytic solution for these measurements was 10^−2^ M DCP. All potential measurements were done with an Orion (720/SA pH/mV meter, Cambridge, MA, USA) at 25 ± 1 °C using all-solid-state potential ISEs”. 

### 2.2. Synthesis of MIPs

“2,4-Dichlorophenol was used as a template for 2,4-dichlorophenol (2,4-DCP). The MIP beads were prepared by the precipitation method [42]. The synthesis process involves 1.0 mmol of DCP (template), 3.0 mmol of MMA (monomer) and 3.0 mmol of EGDMA (cross-linker), placed in a 25 mL capped glass tube. The mixture was left for 1 h for pre-complex formation between 2,4-DCP and MMA molecules then dissolved in 15 mL of acetonitrile. A 80 mg of benzoylperoxide (BPO) were added into the mixture and nitrogen gas was used as a purging gas for 10 min under a gentle flow. In an oil bath at 80 °C, the polymerization process was carried for 18 h. After complete polymerization, the polymer obtained was collected by vacuum filtration, grinded, and washed circularly with 200 mL absolute methanol to remove the un-reacted species. Template removal was carried out using methanol/acetic acid (9:1, *v*/*v*) mixture and then followed by methanolic wash. The obtained polymer was dried overnight at 50 °C under vacuum. In the absence of a template, the non-imprinted polymer (NIP) was also prepared under the same conditions”.

### 2.3. Design and Fabrication of the Screen-printed Electrodes (SPEs) 

**Figure 1:**
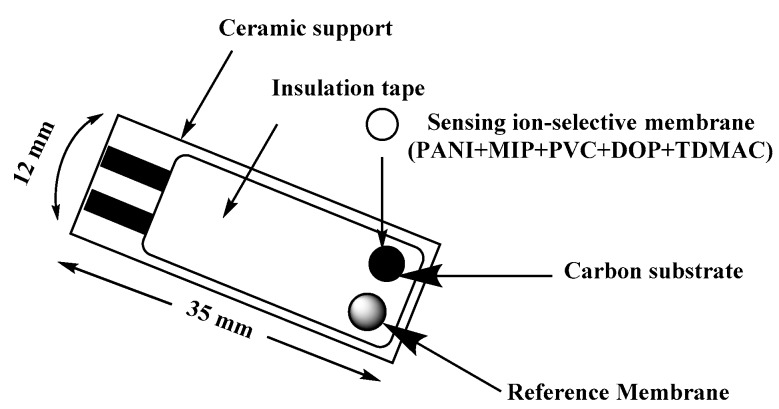
Schematic representation of screen printed planar electrode.

“The design of the screen-printed electrode (SPE) is shown in Figure 1. Both carbon and Ag/AgCl were printed on Alumina substrate of 0.1 mm thickness and 35 mm length. The screen for either carbon or Ag/AgCl ink printing was of 2 mm width. The membrane cocktail of SPEs was prepared by dissolving 100 mg of the components in ~1.5 mL THF as: MIP or NIP beads (9.9 wt %), TDMAC (1.2 wt %), PVC (31.2 wt %), DOP (56.3 wt %) and PANI (1.4%). The reference membrane cocktail was prepared by dissolving 78.1 mg polyvinyl butyral (PVB), 50 mg NaCl in 1 mL methanol. The SPE was rinsed with water and THF before use. 15 µL of the two different membrane cocktails were drop-casting onto the SPE and reference membrane, respectively and left to dry for 6 h”. 

“For comparison, MIP/DCP-ISE were also fabricated from membranes have no PANI, drop-casted on the carbon screen-printed platform. Membrane sensors without PANI were the same as for the respective SPEs. The obtained membranes were of 0.1–0.2 µm thickness. For non-equilibrium potentiometric detection of de-protonated 2,4-DCP, all electrodes were conditioned for at least 2 h in 30 mm Tris buffer (pH 5)”. 

### 2.4. Potentiometric Measurements

“All potentiometric measurements were performed at 20 ± 1 °C using a digital pH/mV meter (Orion SA 720, Cambridge, MA, USA) with the SPE [or Coated wire electrode (CWE)]. All 2,4-DCP concentrations (1.0 × 10^−6^ to 1.0 × 10^−2^ M) were freshly prepared in 30 mm tris buffer (pH5). The electrode potential was measured for each concentration and the resulting EMF values were used for constructing the calibration curve. Selectivity coefficients (*K^p^°^t^_ij_*) were evaluated using the so called “modified separate solution method (MSSM) [43]”.

## 3. Results and Discussions

### 3.1. Characterization of the MIP Particles

**Figure 2:**
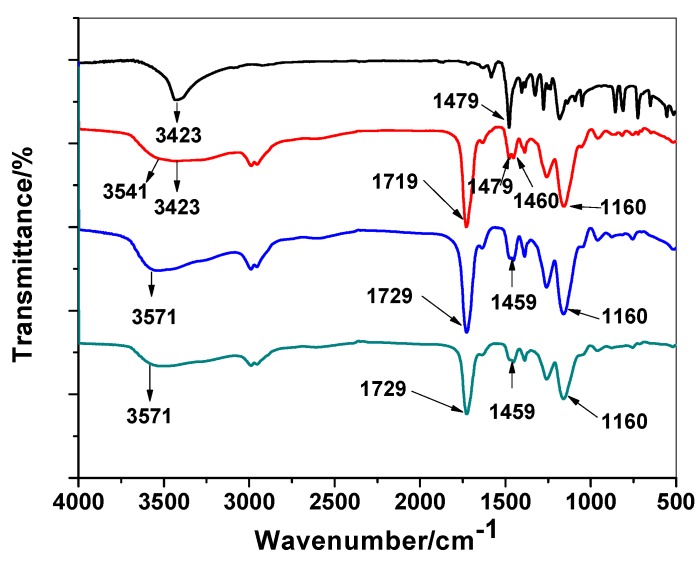
FT-IR spectra for 2,4-Dichlorophenol (DCP), DCP/molecularly imprinted polymers (MIP), MIP/washed and non-imprinted polymer (NIP) beads.

Polymer characterization has been made by FT-IR spectral analysis to show the imprinting process using MMA monomer through the absence/presence of 2,4-DCP on either washed or non-washed polymer beads. As shown in Figure 2, the spectrum of 2,4-DCP showed a strong medium band assigned to stretching O–H band and located at 3423 cm^−1^. In addition, sharp and strong peak around 1479 cm^−1^ which is assigned to aromatic ring stretching vibrations. All these assignable peaks clearly appeared in MIP/MMA before the removal of 2,4-DCP and completely disappeared in the spectrum of MIP particles after 2,4-DCP removal. The IR spectrum of MIP/MMA/EGDMA revealed a weak and broad band at 3543 cm^−1^ for νOH stretching and other peaks at 1719 cm^−1^ and 1160 cm^−1^ for –C=O or –C–O stretches, respectively, which are commonly present in all IR-spectra because of the use of EGDMA cross-linkers. The νC=O in both wasged MIP and NIP beads was located at 1729 cm^−1^. 

“This shift may be attributed to the contribution of the carbonyl group in the interaction with the DCP molecule. The –O–H stretch outcomes from the methacrylic monomer have a broad peak at 3543 and 3423 cm^−1^. This peak appeared at 3571 cm^−1^ in both NIP and washed MIP particles and becomes broader. From all of the above, we can prove the possibility of the interaction between the phenolic O–H group and the carbonyl from the carboxylic group of MMA. This confirms the imprinting process of 2,4-DCP using MMA as a functional monomer”.

**Figure 3:**
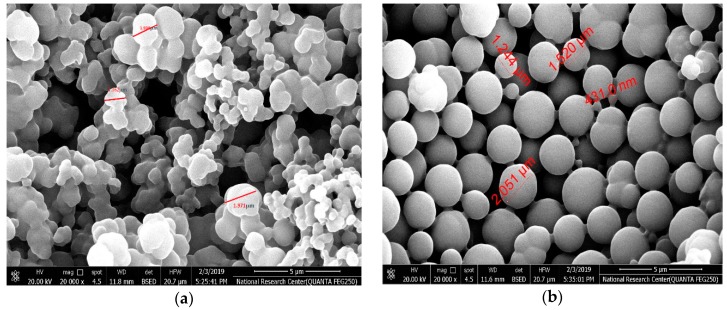
SEM images of (**a**) MIP and (**b**) NIP beads.

“The surface of the resulting imprinted MIP beads was analyzed by scanning electron microscopy (SEM) (JOEL, Osaka, Japan). As seen in Figure 3, the DCP imprinted and NIP beads are uniform and spherical in shape with a diameter distribution of 1.2–1.9 µm and 0.43–1.5 µm, respectively. The difference in particle size between MIP and NIP beads can be attributed to the imprinting effect. Because of this size and spherical shape, the obtained beads can be dispersed well in the ISE membrane and revealed high binding capability towards 2,4-DCP. Because of the well dispersion of the beads in the polymeric membrane, more available binding sites in the ISM and thus result in better response performance can be obtained [44]”.

**Figure 4:**
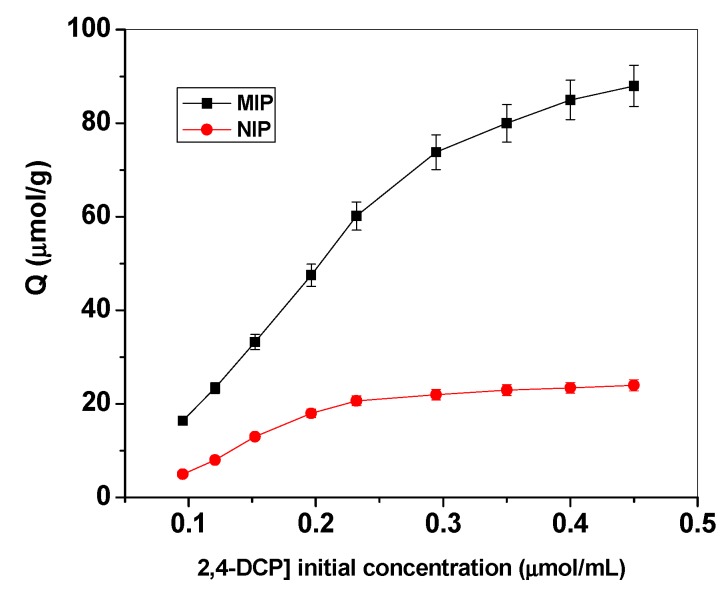
Binding isotherm for MIPs and NIPs. Conditions: 20.0 mg of polymer; t=25 °C; V=10.0 mL.

Binding isotherms and scatchard analyses were performed with different initial 2,4-DCP concentrations at fixed amounts of either MIPs or NIPs micro beads. The mixtures were left until reaching equilibrium and the remaining 2,4-DCP concentrations were evaluated spectrophotometrically at λmax = 284 nm.The resulting binding capacity (Q) of MIPs was calculated according to following Equation (1):

*Q = DCPbound (µmol/mL)/MIP(gm) = (C_i_ – C_f_)V_s_ x 1000/m_MIP_* (1)

where *Q* is binding capacity (μmol/g), *C_i_* the initial DCP concentration (μmol/mL), *C_f_* the final DCP concentration (μmol/ml), *V_s_* the volume of tested solution (mL) and *m_MIP_* the mass of dried polymer (mg). The calculated binding capacities were used to plot the binding isotherms (Figure 4).

**Figure 5:**
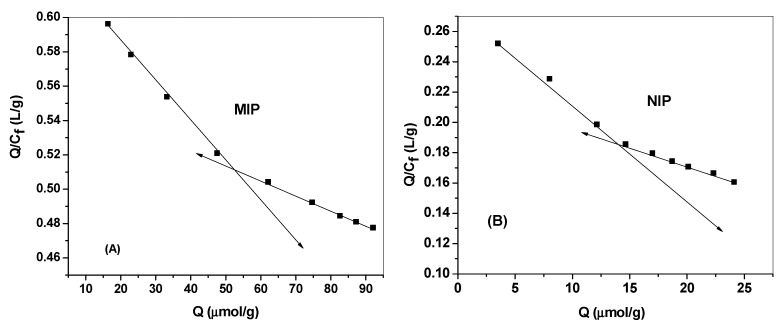
Scatchard plot for (**A**) MIPs and (**B**) NIPs. Conditions: 20.0 mg of polymer; t=25 °C; V=10.0 mL.

The Scatchard analysis was subsequently used to evaluate the binding characteristics using Equation (2):

*Q/C_f_ = (Q_max_-Q)/K_d_* (2)

where *Q* is the binding capacity, *C_f_* is the free DCP concentration at equilibrium (μmol/mL), *Q_max_* is the maximum apparent binding capacity, and *K_d_* is the dissociation constant at binding site. As shown in Figure 5a,b, The *K_d_* and *Q_max_* for the high affinity binding sites were found to be 416.6 μmol/L and 264.3 μmol/g for MIP beads and 163.9 μmol/L and 45.1 μmol/g for NIP beads, respectively.

Imprinting factor (IF) is a useful measure of the presence of the template during polymerization, and it is defined in Equation (3):

*IF = Q_max_ (MIP)/Q_max_ (NIP)* (3)

where *Q_max_ (MIP)* and *Q_max_ (NIP)* are the bound concentrations on the imprinted and the corresponding non-imprinted polymers, respectively. The imprinting factor (IF) is calculated to be 5.86.

### 3.2. Sensor Analytical Features

**Figure 6:**
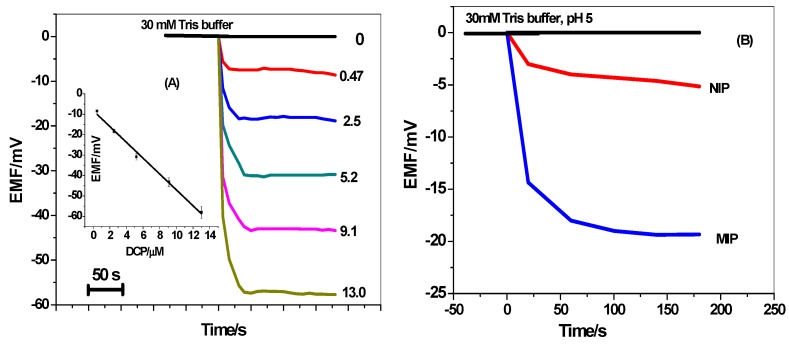
(**A**) The dynamic potentiometric responses of screen-printed platform towards neutral 2,4-DCP in 30 mm Tris buffer at pH 5. The inset shows the measuring calibration plot for 2,4-DCP; (**B**) The potential responses to 2.5 μm neutral 2,4-DCP using the blank, NIP and MIP based membranes.

For a liquid-contact ISE, the membrane sensor is usually conditioned with the primary ion and the potentiometric response is measured under classical equilibrium conditions (30 mM HCO_3_^−^/CO_3_^2−^ buffer, pH 10). In our previous work, it was found that phenols in their neutral forms revealed super-Nernstian anionic responses for polymeric membrane potentiometric sensors containing quaternary ammonium salt and MIPs under near-neutral pH conditions [20,45]. These unexpected super-Nernstian anionic behaviors were clarified on the basis of the net movement of H^+^ ions from the membrane to the aqueous phase. This movement is stimulated by the association of the neutral phenols with quaternary ammonium salt doped in the sensing membrane. According to these, we applied in this work the concept of the non-classical response in 2,4-dichlorophenol (DCP) detection. To ensure the presence of neutral 2,4-DCP, 30 mM Tris buffer solution of pH 5.0 was used as the solution background. To obtain good sensitivity for the detection of neutral DCP, all experiments were done after the optimization of the experimental factors. From the obtained results, it was found that the sensors revealed the best response behavior and performance after using the non-polar plasticizer DOP. This confirms that neutral 2,4-DCP prefers the DOP solvent to be extracted into the ISM. Figure 6a represents the response of the screen-printed platform in absence/presence of DCP under the optimized conditions. The amount of 2,4-DCP is estimated from the difference in potential between the baseline values and those at a fixed time (i.e., 100 s). At higher concentrations of DCP, we noticed stronger anionic response due to the more net movements of H^+^ from the membrane to the aqueous phase [28]. The obtained results indicated that the electrodes revealed a potential response which is directly dependent on the concentration of neutral DCP. The sensor can detect low level concentration of 2,4-DCP down to 0.13 µM within a linearity range starts from 0.47 to 13 µm.

For comparison, the response of NIP-based membrane sensors towards neutral 2,4-DCP was also recorded. As mentioned in Figure 6b, the potential response of MIP-membrane based sensor revealed higher anionic response than the NIP membrane-based sensor towards the same concentration of DCP (i.e., 2.5µm). This can be considered as a proof for the successful imprinting of 2,4-DCP. From all of the data above, we can confirm that low cost screen-printed platforms are an effective alternative to expensive noble metal electrode substrates. 

### 3.3. Selectivity 

**Figure 7:**
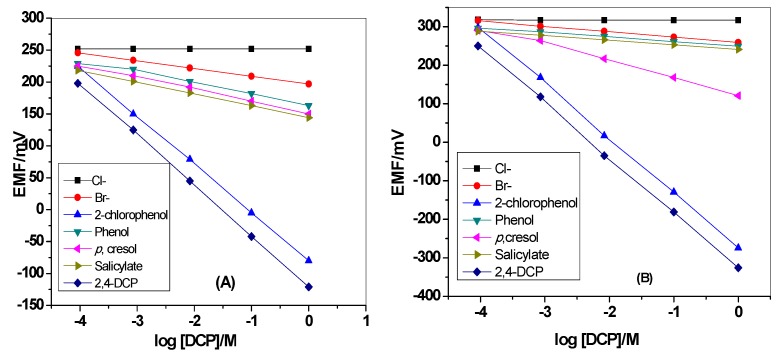
Potentiometric selectivity of MIP membrane-based sensors (**A**) with and (**B**) without polyaniline(PANI) as a solid-contact layer.

The selectivity of the presented electrodes over other neutral phenols such as 2-chlorophenol, phenol, *p*-cresol, salicylic acid, and other common anions such as Br^−^, Cl^−^ is shown in Figure 7. The potential responses towards DCP and other interfering species were measured in 30mm Tris buffer, pH 5. All of the phenols used in the selectivity test have pKa values located within 7.89–13.6 range. This means that at pH 5, they will exist in their un-dissociated forms. The selectivity order for MIP/PANI membrane based sensor is: DCP > 2-chlorophenol > *p*-cresol > salicylate > phenol > bromide > chloride. The effect of PANI layer on the selectivity behavior of the sensor was also tested. The selectivity order for MIP membrane based sensor is: DCP > 2-chlorophenol > phenol > *p*-cresol > salicylate> bromide > acetate> formate > chloride. The selectivity order for neutral phenols is compatible with the lipophilicity in addition to the acidity of these phenols. As previously reported, a phenolic compound that possess high acidity and lipophilicity reveals a stronger super-Nernstian response with an anionic slope [45]. Acid dissociation constants in addition to partition coefficients of 2,4-DCP, 2-chlorophenol, *p*-cresol, salicylic acid and phenol are 7.89, 8.52, 10.3, 13.6 and 9.99, and 3.06, 2.15, 1.94, 2.26 and 1.46, respectively [46]. As noticed, 2,4-DCP has stronger acidity and higher lipophilicity over the other tested phenol derivatives. As shown in Table 1, the sensors revealed higher selectivity over all tested phenolic compounds and other inorganic anions and reflects the importance of using MIP beads as a recognition sensory element. 

### 3.4. Constant-current Chronopotentiometry

**Figure 8:**
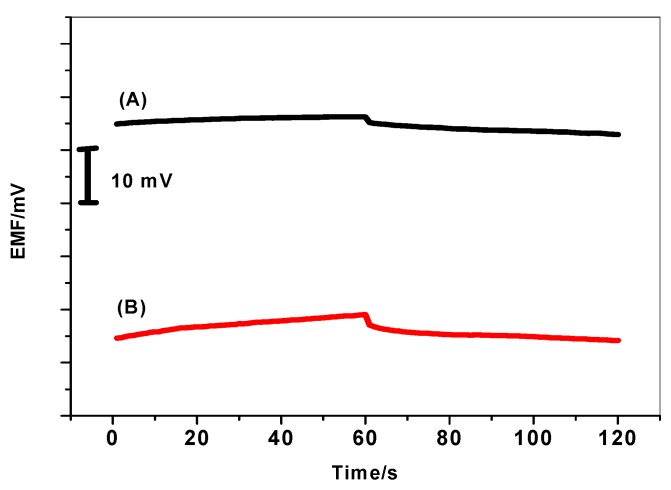
Chronopotentiometry for DCP/MIP-ISEs (A) with and (B) without PANI as a solid contact material.

Short-term potential stability was studied using chronopotentiometric methods. Figure 8 illustrates the typical chronopotentiogram of both MIP/PAN/-ISEs and MIP/ISEs for comparison. The potential drift *(ΔE/Δt*) of MIP/PAN/-ISEs is 20.7 ± 2.2 µV/s (n = 3), which is much lower than that of MIP/ISEs (80.6 ± 3.3 µV/s) (n = 3). This indicates that the potential drift of the electrodes could be minimized by the introduction of PAN directly into the polymeric membrane. The redox capacitance for MIP/PAN/-ISEs is calculated to be 48.1 ± 2.3 µF by using the equation *ΔE/Δt* = I/C [47]. For comparison, the estimated capacitance of MIP/ISEs was found to be 14.7 ± 1.1 µF. From the above results, we can correlate the relationship between the potential stability *(ΔE/Δt*) or capacitance (C_L_) and the presence of PAN as a solid contact material. The bulk resistance (R_b_) of the MIP/ISEs and MIP/PANI/ISEs is found to be about 2.5 ± 0.7 and 0.75 ± 0.08 MΩ, respectively. The difference in total resistances can be explained as the result of PANI solubility in the membrane. 

### 3.5. Electrochemical Impedances and Chronopotentiometry Measurements

**Figure 9:**
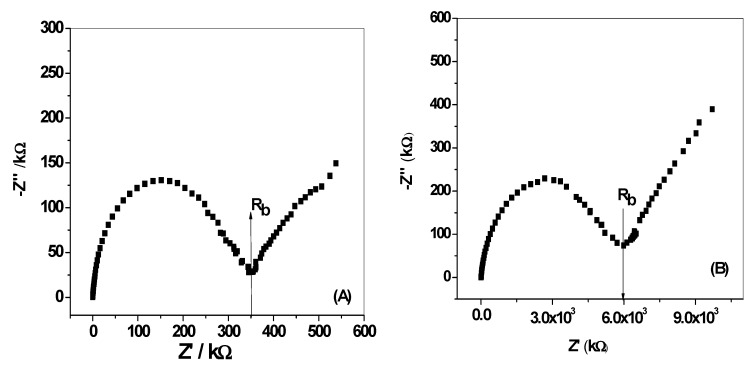
Impedance plot (**A**)for DCP/MIP -ISEs with and, (**B**) without PANI as a solid contact material.

The impedance plots for all-solid-state DCP-ISEs were shown in Figure 9. The bulk impedance of the membrane (*R_b_*) and geometric capacitance (*C_g_*) can be estimated from the high-frequency semicircle. The values of (*R_b_*) and (*C_g_*) were found to be (*R_b_* = 0.35 MΩ, *C_g_* = 43.8 ± 3.2 nF) and (*R_b_* = 6.0 MΩ, *C_g_* = 29.2 ± 2.1 nF) for MIP/PAN/-ISEs and MIP/ISEs, respectively. From the low-frequency branch (semicircle), which is characteristic for the type of solid contact used, the redox capacitance (*C_L_*) is estimated. As shown in Figure 9b, the MIP/ISE presents large semicircle at the low-frequency range. This can be defined to the low capacitance and the high resistance at the “blocked” interface between the sensing membrane and the electronic conductor in the SPE platform. This can explain the long-term potential drift observed for MIP/ISE. For MIP/PAN-ISE, a depressed low-frequency semicircle is obtained and attributed to the presence of PANI layer which is responsible to ion-to-electron transduction through the interface located between the ISM and the conductive substrate. The low-frequency capacitance (*C_L_*) for MIP/ISE and MIP/PAN-ISE were *C_L_* = 4.09 ± 0.6 µF and *C_L_* = 10.8 ± 1.1 µF, respectively.

### 3.6. Water Layer Test

**Figure 10:**
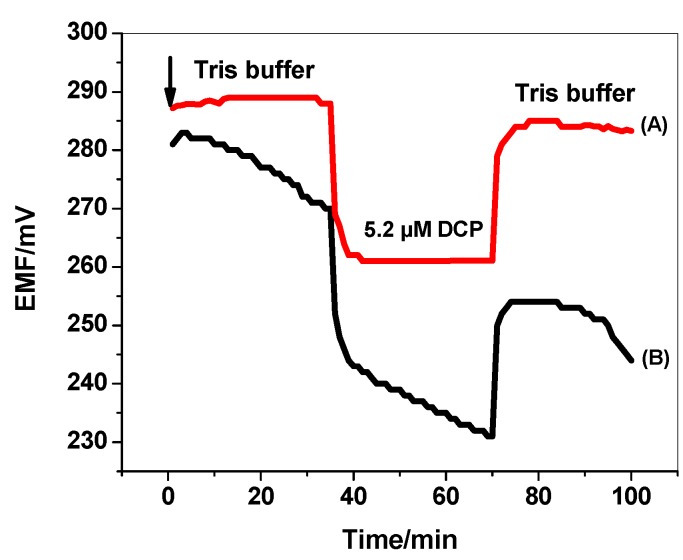
Water-layer tests for the DCP-ISE (A) with and (B) without PANI as the solid contact.

The presence of water film at the interface between the ISM and the electronic conductive substrate can act as a localized microscopic pool of water [48]. It has a prejudicial effect to the potential stability and life time of the electrodes. Therefore, it is important to test the absence of this water layer in the presented ISEs in presence/absence of PANI layer. As shown in Figure 10, a stable potential was observed when MIP/PAN-ISE was immersed in Tris buffer. After the replacement of Tris buffer solution by 5.2 µm DCP solution, again stable potential is observed. After repeating the test with MIP/ISE, an immediate large potential shifts were recorded. It is obvious that a noticeable potential drift is recorded for MIP/ISE when changing back from tris buffer to 2,4-DCP. This indicates that a water film was formed between the ion sensing membrane and the electronic conductor. However, this drift is completely disappeared when using the PANI as the solid-contact layer. This demonstrates that the water layer was also eliminated by using the highly hydrophobic PANI layer.

### 3.7. Analytical Applications

There are different causes for the presence of chlorophenols in drinking-water. They can be released in water as a result of the chlorination of phenols during disinfection. In addition, they can be produced as by-products after the reaction of phenols with hypochlorite or as degradation products of phenoxy herbicides. Chlorination increased the concentrations of 2-chlorophenol (2-CP), 2,4-dichlorophenol (2,4-DCP) and 2,4,6-trichlorophenol (2,4,6-TCP) [49]. The levels of chlorophenols in drinking-water are quite low and vary from one location to another. The maximum admissible level in water was regulated by the European union (EU) and US Environmental Protection Agency (EPA), and found to be 0.5 µg/L [11]. The proposed MIP-based potentiometric sensor was introduced in a potentiometric setup and used to assess 2,4-DCP in different water samples collected from different places around Cairo, Egypt. The samples were then spiked with different concentration levels of 2,4-DCP and finally analyzed using the standard addition method. The accuracy was checked by measuring the recoveries of DCP in the water samples. The results were presented in Table 2. It can be seen that the recoveries of water samples vary from 95.8% to 108.3%. This can prove the feasibility of the proposed sensors for the assessment of 2,4-DCP in different complex samples. 

## 4. Conclusions

Cost-effective and reliable disposable solid contact screen-printed electrodes based on uniform-sized MIP beads for 2,4-DCP detection have been presented. Polyaniline (PANI) was used as the ion to-electron transducer and showed excellent conductivity. The presented screen-printed platforms exhibited a rapid potential response towards 2,4-DCP in its neutral form with high selectivity over other phenol derivatives, in addition to high accuracy and precision. The overall presented method is precise, accurate, and inexpensive regarding reagent consumption and the equipment involved. The sensors were successfully applied to 2,4-DCP determination for concentration levels down to 0.4 µm. 

## Figures and Tables

**Table 1:** Selectivity coefficients, *Log K^p^°^t^_DCP,J_*, of the proposed screen-printed sensors.

Sensor	*Log K^p^°^t^_DCP,J_*					
	Cl^−^	Br^−^	2-chlorophenol	phenol	*p*-cresol	salicylate
MIP	−10.8	−9.9	−0.8	−9.7	−7.5	−9.6
MIP+PANI	−6.3	−5.4	−0.7	−2.0	−4.4	−4.5

**Table 2:** Application of the proposed sensor to determination of DCP in the water samples.

Sample	Proposed Sensor, (µM)*	Amount, µM *			HPLC, (µm) * [49]
		Added	Found	Recovery, (%)	
1	0.15 ± 0.06	0.5	0.67 ± 0.04	103.1	0.17 ± 0.06
2	0.22 ± 0.03	0.5	0.78 ± 0.07	108.3	0.21 ± 0.03
3	0.18 ± 0.02	0.5	0.65 ± 0.02	95.5	0.16 ± 0.02
4	0.31 ± 0.04	0.5	0.78 ± 0.08	96.2	0.35 ± 0.04
5	0.42 ± 0.03	0.5	0.95 ± 0.05	103.2	0.45 ± 0.03

* Average of three measurements ± standard deviation.
